# A middle mesenteric artery

**DOI:** 10.1007/s00276-012-0987-y

**Published:** 2012-07-22

**Authors:** Stanislaw Milnerowicz, Artur Milnerowicz, Renata Taboła

**Affiliations:** 1 Department of Gastrointestinal and General Surgery, Wrocław Medical University, 66 Curie-Skłodowska Street, 50-369 Wrocław, Poland; 2 Department of Vascular Surgery, Wrocław Medical University, 213 Borowska Street, Wrocław, Poland

**Keywords:** Anatomical variation, Middle colic artery, Middle mesenteric artery

## Abstract

In 114 cases of the transverse colon isolated from cadavers (50 male, 64 female), anatomical examinations of the arterial system of the colon were performed. Arteriograms were obtained after dissecting and contrasting the colonic vessels with Mixobar contrast. In one case, on arteriography of the colon with its mesentery isolated from a 55-year-old male cadaver, a rare anatomical variant was found. The third mesenteric artery originated directly from the aorta—halfway between the superior and inferior mesenteric arteries and ascended obliquely in the direction of the hepatic flexure of the colon. Supply area of the artery was typical for the middle colic branch of the superior mesenteric artery: the distal segment of the ascending colon and the transverse colon. Such a variation, although very rare, may have particular impact on diagnosis and even the method and range of surgery.

## Introduction

In anatomical studies based on large material of over 1,000 colonic dissections, the middle colic artery is described as a variable artery stemmed off the superior mesenteric artery and supplying mainly the transverse colon [3, 5, 8]. The term concerns not only its structure, but also the range of its delivery. In 3–5 % of the cases the middle colic artery is absent [5]. Rarely anatomical studies have detected variations in the middle colic artery origin. The artery is thought to be colonic artery when it arises from the celiac trunk or its branches. The term middle mesenteric artery is reserved for the vessel directly originating from the aorta between the superior and inferior mesenteric arteries. Origin of the middle colic artery from the celiac trunk was first described by Tandler [10]. Arterial variation of the colon found during our study occurs extremely rarely. Its incidence has been established <0.1 % [3]. So far only a few authors have reported the artery supplying the transverse colon that directly originates from the aorta instead of the superior mesenteric artery. Its peripheral course and segment of the intestine fed varies from case to case. The nomenclature also differs depending on the author: Pillet [6] proposed the term artère mèsentèrique moyenne, Delannoy [2] named it artère mèsenterique supèrieure double, while Benton and Cotter [1] described the variation as duplication of inferior mesenteric artery. The artery presented in this paper arose halfway between the superior and inferior mesenteric arteries and supplied typical area for the middle colic branch of the superior mesenteric artery: the distal segment of the ascending colon and the transverse colon.

## Materials and methods

**Fig. 1:**
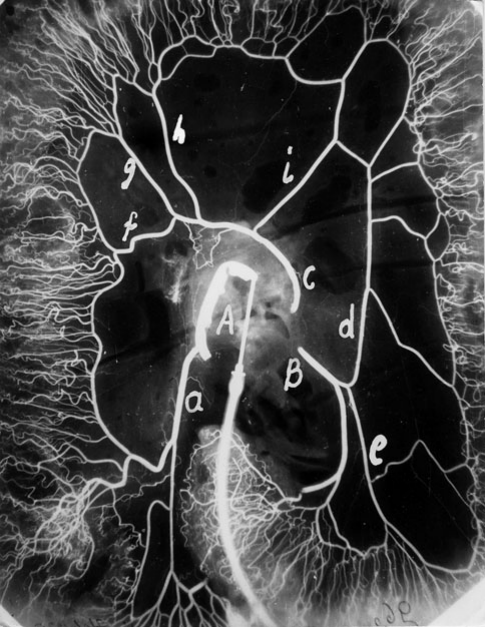
Arteriogram of the arteries of the large intestine with the catheter positioned in the superior mesenteric artery. *A* superior mesenteric artery, *B* inferior mesenteric artery, *C* middle colic artery (originates directly from the aorta), *a* ileo-colic artery, *d* left colic artery, *e* sigmoid artery arising from the left colic artery. Branches of the middle colic artery: *f* right colic artery, *g* right middle colic artery, *h* median middle colic artery, *i* left middle colic artery

The studies were carried out on 114 (50 male and 64 female) colons isolated from human bodies during autopsy. In all the cases arteriography of colonic arteries was performed. After dissecting the aorta, the catheter was introduced to the superior mesenteric artery and Mixobar (1:3 aqueous solution of Astra-Sweden) was injected to show the area of blood supply for the artery (Fig. 1). In a few cases, contrast was additionally introduced by inferior mesenteric artery.

Radiographs of the specimens were made by 65 kV/10 mA device, from 1 m distance to obtain natural size pictures. Exposition time varied between 0.4 and 0.8 s, according to the thickness of the object.

## Results

The number of arteries arising out of the superior mesenteric artery supplying the colon and their anatomical nomenclature is presented in Table 1. In our material, the middle colic artery branched off the superior mesenteric artery in 99.12 % of the cases.

**Table 1:** Branching pattern of superior mesenteric artery to the colon in our material

Variation observed (arteries stemming from superior mesenteric artery)	Number of arteries originating from superior mesenteric artery	Number of cases (%)
Ileo-colic, middle colic artery	2	65 (57.02)
Ileo-colic, right colic, middle colic artery	3	23 (20.17)
Ileo-colic, right colic, middle colic, accessory middle colic artery	4	12 (10.53)
Ileo-colic, right colic, middle colic, accessory middle colic, accessory right colic artery	5	2 (1.75)
Ileo-colic artery	1	1 (0.88)

In one case (0.88 %), during dissection of a 55-year-old cadaver, we found the middle colic artery, which did not stem from the superior mesenteric artery, instead it aroused directly from the aorta, halfway between the superior and inferior mesenteric arteries. The middle mesenteric artery originated from the ventral wall of the aorta and ascended obliquely in the direction of the hepatic flexure of the colon.

Arteriography confirmed the variation, the middle colic artery directly aroused from the aorta. The superior mesenteric artery of 0.8 cm diameter originated from the antero-lateral wall of the aorta. The inferior mesenteric artery of 0.4 cm diameter branched off 5 cm below it. Between them, in the middle (2.5 cm) distance to the left, another very well developed artery of 0.4 cm diameter stemmed from antero-lateral wall of the aorta. The artery ran in front of the aorta, crossing the mesentery slightly to the right. The arteriogram of the middle mesenteric artery is presented in Fig. 1. Its delivery area was typical for the middle colic artery. It was 10 cm long and gave four branches: right colic and three middle colic arteries (right, median and left). Their diameters were similar and measured about 2.5 mm. Anastomoses among them were well developed and their diameters ranged between 1.4 and 1.5 mm. The right colic artery arising after 5.6 cm, delivered left hemicolon up to the splenic flexure. The right middle colic branch originated 8.8 cm from the artery origin, remaining about 10 cm from its beginning. The right and median middle colic branches supplied the right hemicolon and the hepatic flexure. Branches of the left middle colic artery ran to the distant half of the ascending colon. The superior mesenteric artery gave only one branch: the ileo-colic artery, diameter of which was also about 0.4 cm and its length reached 5.7 cm. This artery gave a well-developed branch of the colic artery that gave arteries straight to the coecum and the ascending colon that did not connect with each other. The branch was anastomosed by 1.7 mm diameter vessel with the branch of the middle mesenteric artery.

## Discussion

Fetal ascending colon morphogenesis begins from the fourth month. At this time primitive metameric intestinal arteries of two types, paired and unpaired, start their development. The unpaired visceral branches are to distribute to the gut. They regress and anastomose usually to three main trunks in later stage of an embryo development. The right hemicolon is delivered by branches of the upper mesenteric artery that originates from midgut. Inferior mesenteric artery supplies the hindgut [10, 11]. The middle mesenteric artery in our case is an example of additional unpaired mesenteric artery supplying the midgut and the hindgut. Its direct origin from the aorta and hypothetical embryologic genesis might explain use of the term middle mesenteric artery in the anatomical nomenclature, especially if the middle colic artery, which is thought to be an intermesenteric artery supporting the blood supply from the superior mesenteric artery to the colon, is absent (Fig. 1). The typical anatomical range of delivery for the middle colonic artery: distal part of the ascending colon and the transverse colon made us tend to name the variation the middle mesenteric artery.

To our knowledge, a simple middle mesenteric artery which is typical for the middle colic artery supplying area, even in large anatomical studies on colonic arteries is a rare event [3–5, 7].

So far described cases of additional intestinal arteries arising from aorta and their range of delivery have varied, and the anatomical nomenclature also differs from study to study. Dellannoy [2] during necropsy found two arteries arising from the aorta that fed the small intestine segments and the right and transverse colon. He considered them duplicated superior mesenteric artery: one was the superior mesenteric artery dividing into jejunal and ileal branches and the other one, which supplied the right and transverse colon, represented the middle mesenteric artery. Middle colic artery was absent similarly to our case.

Though additional superior mesenteric artery of similar supplying area—the ascending and the transverse colon, Pillet [6] described as middle mesenteric artery.

Benton and Cotter [1] presented accessory aortic artery supplying the transverse colon and superior portion of the descending colon. In their case middle colic artery was also absent.

Such a variation, although very rare, may have a practical impact on diagnosis or even method and range of surgical treatment [5, 9]. Wider and wider use of conventional angiography, selective angiography, CT and MDCT angiography allows for identifying such a variation before schedule or urgent surgery. The middle mesenteric artery ought to be considered if any of the main branches of the celiac trunk, superior or inferior mesenteric arteries are not visualized. This rare condition discovered on angiography due to abdominal aortic aneurysm repair may change a method from endovascular to conventional surgery in order to prevent colon ischemia. The variant, as a source of blood supply for various segments of colon, must be kept in mind in para-aortal lymphadenectomy.
